# A Comprehensive Benchmark Study of Multiple Sequence Alignment Methods: Current Challenges and Future Perspectives

**DOI:** 10.1371/journal.pone.0018093

**Published:** 2011-03-31

**Authors:** Julie D. Thompson, Benjamin Linard, Odile Lecompte, Olivier Poch

**Affiliations:** Département de Biologie Structurale et Génomique, IGBMC (Institut de Génétique et de Biologie Moléculaire et Cellulaire), CNRS/INSERM/Université de Strasbourg, Illkirch, France; J. Craig Venter Institute, United States of America

## Abstract

Multiple comparison or alignmentof protein sequences has become a fundamental tool in many different domains in modern molecular biology, from evolutionary studies to prediction of 2D/3D structure, molecular function and inter-molecular interactions etc. By placing the sequence in the framework of the overall family, multiple alignments can be used to identify conserved features and to highlight differences or specificities. In this paper, we describe a comprehensive evaluation of many of the most popular methods for multiple sequence alignment (MSA), based on a new benchmark test set. The benchmark is designed to represent typical problems encountered when aligning the large protein sequence sets that result from today's high throughput biotechnologies. We show that alignmentmethods have significantly progressed and can now identify most of the shared sequence features that determine the broad molecular function(s) of a protein family, even for divergent sequences. However,we have identified a number of important challenges. First, the locally conserved regions, that reflect functional specificities or that modulate a protein's function in a given cellular context,are less well aligned. Second, motifs in natively disordered regions are often misaligned. Third, the badly predicted or fragmentary protein sequences, which make up a large proportion of today's databases, lead to a significant number of alignment errors. Based on this study, we demonstrate that the existing MSA methods can be exploited in combination to improve alignment accuracy, although novel approaches will still be needed to fully explore the most difficult regions. We then propose knowledge-enabled, dynamic solutions that will hopefully pave the way to enhanced alignment construction and exploitation in future evolutionary systems biology studies.

## Introduction

Evolutionary theory provides a unifying framework for analysing genomics data and for studying various phenomena in molecular, cell, or developmental biology [1]. Thus, evolutionary-based inference systems are playing an increasingly important role in diverse areas, such as elucidation of the tree of life [2], studies of epidemiology and virulence [3], drug design [4], human genetics [5], cancer [6] or biodiversity [7]. Essential prerequisites for such evolutionary-based studies are the multiple sequence alignment (MSA) and its subsequent analysis [8], [9], [10]. By placing the sequence in the framework of the overall family, MSAs can be used to characterise important features thatdetermine the broad molecular function(s) of the protein, such as the 3-dimensional structure or catalytic sites, and that have been conserved throughout evolution.However, most proteins act in complex, dynamic networks that are dependent on the biological context, for example subcellular localisation, temporal and spatial expression patterns, or environment. Here, MSAs will alsohave a crucial role to play in identifying the specific features, also known as “specificity determining positions” (SDPs), that modulate a protein's function in a given context, for example, interaction domains, regions or sites, targeting signals in the different cell machineries, pathways orcompartments, or post-translational modification sites(phosphorylation, cleavage, etc.) [11], [12], [13].

MSA algorithms have been an active area of research since the 1980s. Traditionally the most popular approach has been the progressive alignment procedure [14], which exploits the fact that homologous sequences are evolutionarily related. A multiple sequence alignment is built up gradually using a series of pairwise alignments, following the branching order in a phylogenetic tree. A number of different alignment programs based on this method have been developed, includingboth global and local approaches. A global MSA algorithm is defined here as one that tries to align the full length sequences from one end to the other. Once the global alignment has been constructed, other methods are often used to identify the more conserved or reliable regions within the alignment. A local algorithm attempts to identify subsequences sharing high similarity. The unreliable or low similarity regions are then either excluded from the alignment, or are differentiated, for example, by the use of upper/lower case characters. Comparisons of many of these methods based on ‘gold standard’ benchmarks [15], [16] showed that none of the existing algorithms were capable of providing accurate alignments for all the test cases. As a consequence, iterative algorithms were developed to construct more reliable multiple alignments, using for example iterative refinement strategies [17], Hidden Markov Models [18] or Genetic Algorithms [19]. These methods were shown to be more successful at aligning the most conserved regions for a wide variety of test cases, although some accuracy was lost for distantly related sequences, in the ‘twilight zone’ of evolutionary relatedness [20], [21].

In the post-genomic era,the growing complexity of the multiple alignment problem has lead to the development of novel methods that use a combination of different alignment algorithms [22], [23], [24], [25] or that incorporate biological information other than the sequence itself [26], [27]. A number of specific MSA problems have also been addressed by programs such as POA [28] for the alignment of non-linear sequences or PRANK [10] for the detailed evolutionary analysis of more closely related sequences. These new MSA construction methods are generally evaluated using one or more alignment benchmarks, for example, BAliBASE [15], OxBench [29] or PREFAB [24], and it is clear that this benchmarking has had a positive effect on their development [30]. Most of the widely used MSA benchmarks were compared in [21] and are also discussed in [31]. The use of objective benchmarks leads to a better understanding of the problems underlying poor performance, by highlighting specific weak points or bottlenecks. Thus, benchmarking can help the developer improve the performance of his software. In turn, the software improvements imply that the benchmarks must continually evolve, if they are to represent the current problems and challenges in the domain [31].

Today, new high throughout biotechnologies are providing us with enough data to build complete evolutionary histories of large sets of genes [32]. For the first time, it will be possible to compare sequences from hundreds of diverse organisms, both present and extinct, to perform detailed studies of the evolutionary patterns and forces that shaped extant genes and to reconstruct the genetic changes that are responsible for the phenotypic differences between organisms. Although the current flood of data clearly provides unique opportunities for systems-level studies, it also poses many new challenges, in addition to the obvious scalability issues. First, although the range of organisms studied has increased recently, a relatively small number of model organisms still dominate the public databases. Second, the protein families represented in today's sequence databases are often more complex, with multidomain architectures, large unstructured (natively disordered) regions, numerous splicing variants, etc. Third, the new sequences are mostly predicted by automatic methods and thus, contain a significant number of sequence errors [33], [34]. For example, the EGASP assessment of gene prediction algorithms showed that the best gene prediction systems are able to predict entirely correct sequences for protein transcripts in the human genome only 50% of the time [35]. The problem has been further exacerbated by the next generation (massively parallel) DNA sequencing instruments that can sequence up to one billion bases in a single day at low cost [36]. These new technologies produce read lengths as short as 35–40 nucleotides, resulting in fragmentary protein sequences that pose problems for bioinformatics analyses [37].If MSA methodology is tokeep pace with the new challengespresented by this complex and often ‘noisy’ sequence data, the alignment benchmarks used for evaluation must now evolve to reflect this changing biological sequence space.

Here, we describe a new protein sequence alignment benchmark designed to reproduce today's sequence exploration requirements and a comprehensive assessment of the performance of some of the most popular MSA programs. Our study was motivated by two major observations. First, most of the existing MSA benchmarks - and as a consequence, most MSA construction algorithms - have focused on the patterns conserved in the majority of the sequences and not enough attention has been paid to the less frequent patterns, or SDPs, that might indicate subfamily-specific or context-specific functions. Second, current MSA programs for protein sequences generally model globular domain structure and evolution. Nevertheless, many proteins, particularly in eukaryotes, are unstructured (natively disordered) or contain large unstructured regions.These regions frequently contain motifs, such as signalling sequences or sites of posttranslational modifications, that are involved in the regulatory functions of a cell [38], [39]. While this complexity alone represents a significant challenge for today's MSA algorithms, another major goal of our study was to investigate the effect of the ‘noisy’ data, including fragmentary or otherwise erroneous sequences, on MSA program performance.

Our benchmark, representing 218 large, complex protein families, has been incorporated in the BAliBASE benchmark suite and provides a complementary test to the existing reference sets. While the previous sets included mainly alignments of shared, structured domains, the reference set described here focuses on (i) subfamily specific features, (ii) motifs in disordered regions, (iii) the effect of fragmentary or otherwise erroneous sequences on MSA quality. The new benchmark tests were then used to evaluate the quality of the alignments produced by some of the most widely used programs for MSA construction. This comparative study allowed us to evaluate the recent progress achieved and to highlight a number of specific strengths and weaknesses of the different approaches. Finally, we propose new directions for the future development of multiple alignment construction and analysis methods.

## Results

### Benchmark alignments

**Figure 1:**
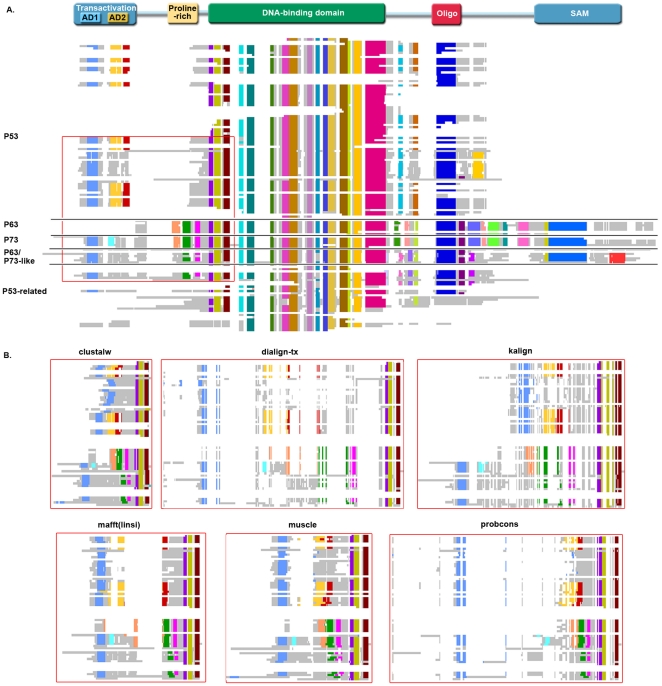
An example benchmark alignment. (A) Reference alignment of representative sequences of the p53/p63/p73 family, with the domain organization shown above the alignment (AD: activation domain, Oligo: oligomerization, SAM: sterile alpha motif). Colored blocks indicate conserved regions. The grey regions correspond to sequence segments that could not be reliably aligned and white regions indicate gaps in the alignment. (B) Different MSA programs produce different alignments, especially in the N-terminal region (boxed in red in A) containing rare motifs and a disordered proline-rich domain.

**Figure 2:**
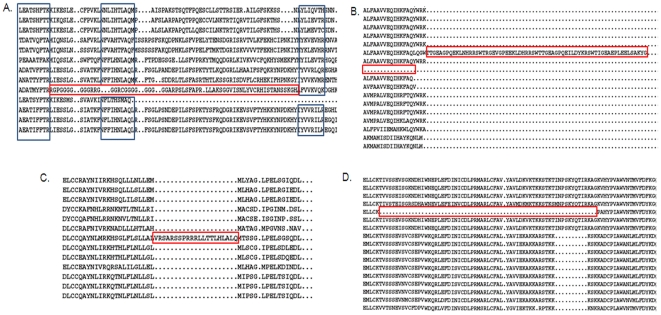
Examples of sequence discrepancies detected. Four types of sequence discrepancies are identified and highlighted by red boxes in the subfamily alignments. A. Potential mispredicted exons are predicted based on the scores of the conserved core blocks (blue boxes) in the subfamily alignment. Here, the ninth sequence contains a segment ‘outlier’ that scores below the defined threshold for the central core block. The region of the sequence identified as a discrepancy is extended to the nearest core blocks in which the sequence is correctly aligned. B. Potential start and stop site errors are predicted based on the distribution of the positions of the N/C-terminal residues. C. Identification of a potential inserted intron, based on the presence of a single sequence with the insertion in a given subfamily. D. Identification of a potential missing exon, based on the presence of a single sequence with a deletion in a given subfamily.

The BAliBASE benchmark suite contains multiple sequence alignments, organised into 9 Reference Sets representing specific MSA problems, including small numbers of sequences, unequal phylogenetic distributions, large N/C-terminal extensions or internal insertions, repeats, inverted domains and transmembrane regions. Here, we have constructed a new BAliBASE test set, Reference 10, composed of 218 reference alignments and containing a total of 17892 protein sequences, which were obtained using a query-based database search protocol. Details of the benchmark alignments are provided in the Methods section. For each reference alignment, we then identified the locally conserved regions, or ‘blocks’, using an automatic method. This led to the definition of 9131 blocks, covering on average 46% of the total multiple alignment. The remaining regions of the reference alignments, corresponding to the unalignable or unstable segments, were excluded from the analyses performed in this work. The resulting benchmark alignments reflect some of the problems specific to aligning large sets of complex protein sequences. For example, many of the protein families (>64% of the alignments) have multidomain architectures and their members often share only a single domain. Another important feature of the alignments is linked to the distribution of the conserved blocks. The alignment of the highly studied P53/P63/P73 family (Figure 1A), illustrates this conceptwith only 18% of the blocks present in most (>90%) of the aligned sequences, while 30% are found in less than 10%. These ‘rare’ segments or patterns are often characteristic of context-specific functions, e.g. substrate binding sites, protein-protein interactions or post-translational modification sites.Finally, the alignments have a high proportion of sequences with ‘discrepancies’,i.e. unexpected or discordant extensions, insertions or deletions, as shown in Figure 2. These discrepancies may correspondto naturally occurring variants or may be the result of artifacts, including PDB sequences (typically covering a single structural domain), proteins translated from partially sequenced genomes or ESTs, or badly predicted protein sequences.In the alignment in Figure 1A, 45% of the aligned sequences (61 out of 134) contain one or more of these discrepancies.

### MSA program evaluation: overall alignment quality

**Figure 3:**
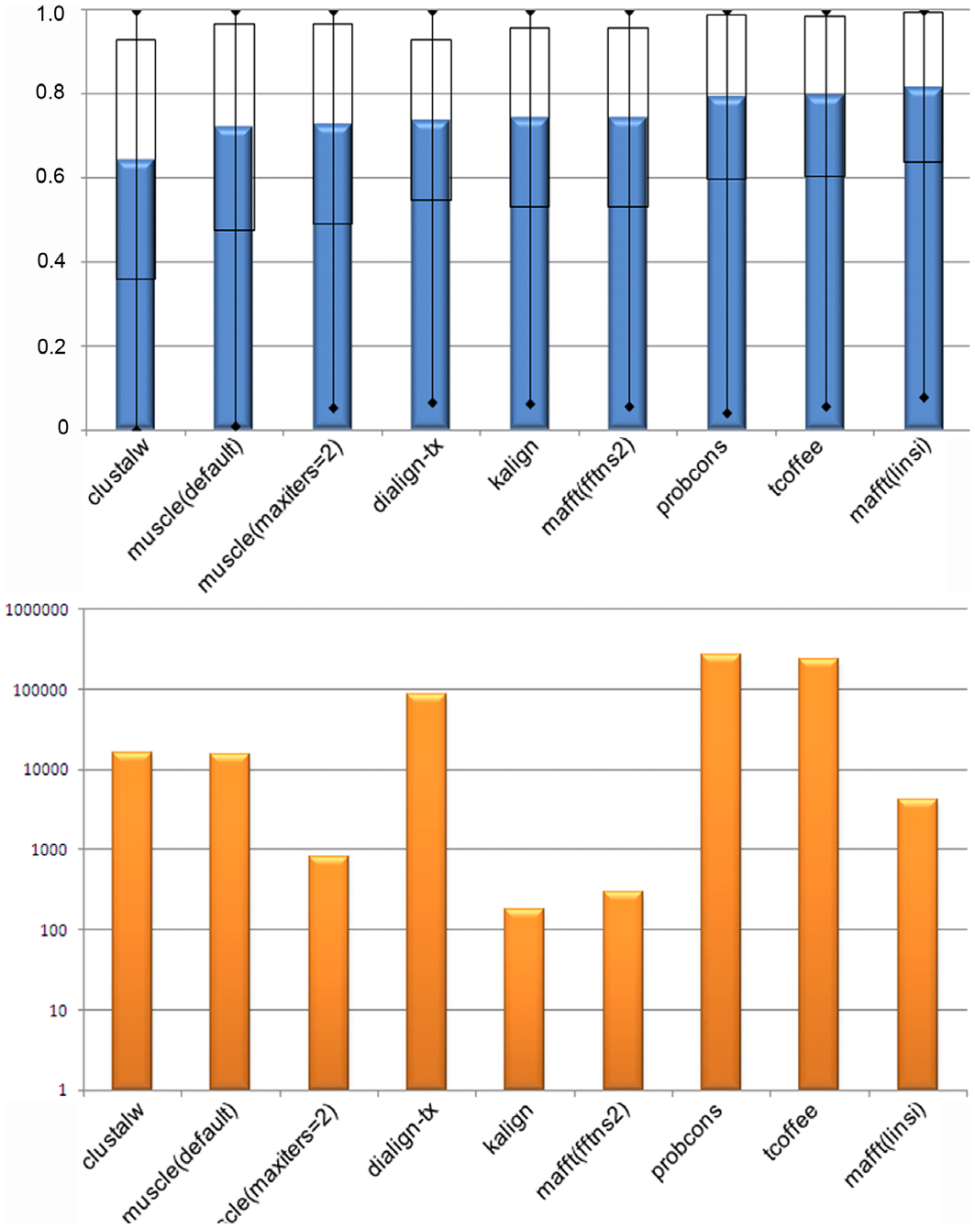
Overall alignment performance for each of the MSA programs tested. (A) Overall alignment quality measured using CS. Programs are shown ranked by increasing quality scores. Error bars correspond to one standard deviation.(B) Total run time for constructing all alignments (a log10 scale is used for display purposes).

For each of the 218 reference alignments in the benchmark, we applied eight alignment programs, resulting in a total of 1744 automatically constructed MSAs. The overall quality of these automatic alignments was measured using the Column Score (CS) described in Methods. This initialexperiment generally confirmed previous findings, in terms of program ranking (Figure 3). Probcons, TCoffeeand the most recent version of Mafft (linsi) (version 6.815) achieved the highestaverage scores (79.4% and 81.6% respectively).Nevertheless, Probcons and TCoffee took over 2.7 days to compute all the alignments, while Mafft (linsi) took 1.2 hours.The fastest program, Kalign, required only 3.0 minutescomputation time, although some loss of accuracy was observed (74.3%). As expected, the more recent methods incorporating both local and global algorithms were generally more accurate than older methods, based on global (ClustalW: 64.4%) or local (Dialign-tx: 73.8%) algorithms alone. Individual alignment accuracy was highly variable even for the best programs (with a standard deviation of 19.6, 19.1and 18.9 for Probcons, TCoffee and Mafft (linsi) respectively). This is in agreement with previous observations showing that some alignments are more difficult than others [10], [20].

**Figure 4:**
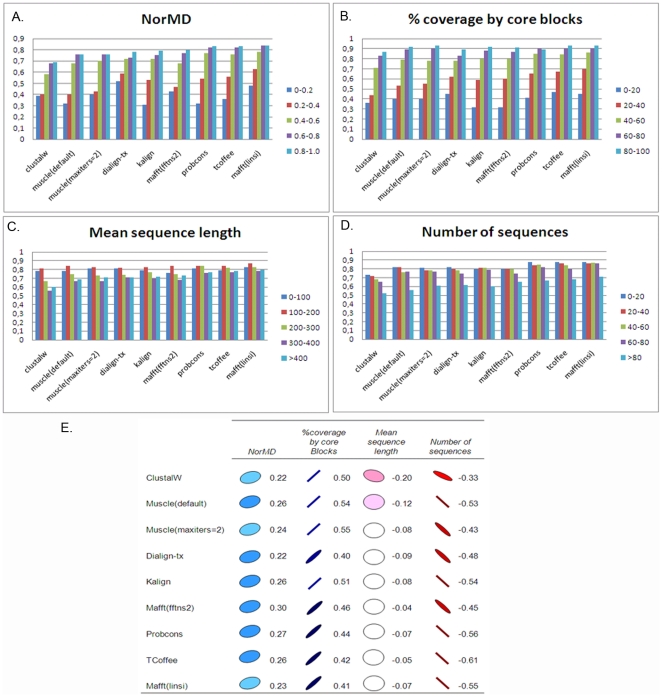
Factors affecting overall alignment quality. Average alignment quality scores (CS) for each MSA program tested and for eachglobal alignment attribute:(A) CS versus NorMD, (B) CS versus the percentage of the alignment covered by the blocks, (C) CS versus mean sequence length, (D) CS versus the total number of sequences.(E) Pearson correlation coefficients of overall quality scores (CS) for each program with global alignment attributes (blue: positive correlation, red: negative correlation).

To investigate in more detail the factors affecting the performance of each program, we characterized each alignment using a number of ‘global’ attributes describing the overall full-length alignment, including the number of sequences to be aligned, their length, an MSA objective function (norMD) [40] and the percentage of the alignment covered by the blocks. Figure 4(A–D) shows the distributions of the overall alignment quality scores obtained by each MSA program for each global attribute. These distributions, together with a correlation analysis (Figure 4E), showed that more closely related sequences were generally aligned better (positive correlation for all programs with the norMD and percent coverage by blocks), as might be expected. For the more difficult alignment tests, e.g. with norMD<0.2, the mean CS scores were less than 0.5 for all the aligners included in this study. The length of the sequences had less effect on alignment quality, although longer sequences tended to be less well aligned. In contrast to some previous studies [20], [21], we observed a negative correlation with the number of sequences in these alignments, i.e. the alignments with a larger number of sequences were less well aligned. For alignments with more than 80 sequences, only Mafft (linsi) achieved CS scores higher than 0.7.

### Effect of sequence discrepancies on alignment quality

To study the effect of the new sequences resulting from high throughput biotechnologies, we identified sequence discrepancies that might be due to fragmentary or erroneous sequences using an empirical rule-based approach (described in Methods). The method exploits information from the reference alignments to classify the sequences in each alignment into a number of subfamilies and to construct a representative model for each protein subfamily, including characteristic conserved blocks and typical start/stop sites. Each subfamily sequence was then compared to the model in turn, in order to identify ‘outlier’ sequences, with one or more discrepancies. The discrepancies we considered included: (i) divergence of the sequence from conserved core blocks that might indicate badly predicted exons, (ii) insertions that may be due to introns predicted to be coding, (iii) deletions that may be due to missing exons and (iv) potential start and stop site mispredictions. Although the method used here to detect sequence discrepancies may also identify a number of naturally occurring proteins, such as splicing variants, our main goal was to construct a set of reliable sequences for use in the following experiments.

**Figure 5:**
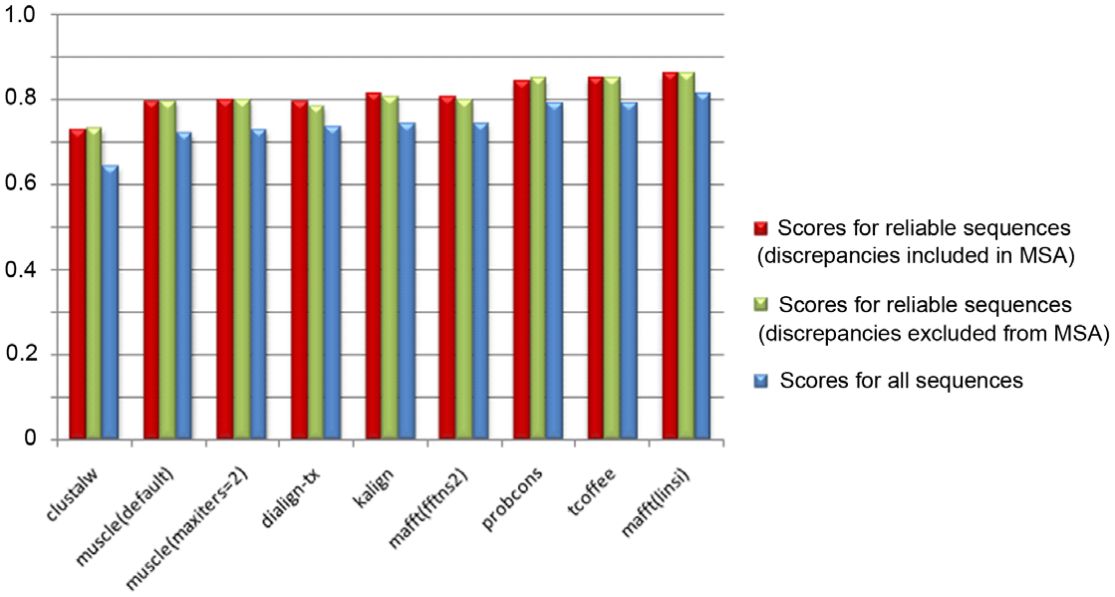
Comparison of alignment quality scores for sequence sets with and without potential error sequences. Quality scores (CS) for alignment of reliable sequences when discrepancies are included in the alignment set are shown in red. Quality scores for the same set of sequences when discrepancies are removed from the alignment set are shown in green. Scores for all sequences (from figure 2) are shown (in blue) for comparison purposes.

In the first experiment, all the sequences (the reliable sequences and those with discrepancies) were used as input for each MSA program. The alignment quality scores were then calculated based only on the reliable sequences (ignoring the sequences with discrepancies) and compared to the scores obtained in the previous test for all sequences (Figure 5). Significant differences (one-tailed student t-test)were observed for all the MSA programs tested, implying that sequences with discrepancies are aligned less well than reliable sequences.

In the second experiment, the sequences with discrepancies were excluded from the benchmark test sets and each sequence set was realigned using the eight MSA programs.The quality of the resulting alignments was again measured using the CS score (Figure 5). No significant differences were observed for the alignment scores based on the reliable sequences, when sequences with discrepancies were included or excluded from the MSA.

Based on these two experiments, we conclude that the MSA programs tested are capable of accurately aligning the reliable sequences, even in the presence of a large proportion of sequences with discrepancies. Nevertheless, it is important to note that, in the presence of sequences with discrepancies, the subsequent exploitation of the MSA and in particular the identification of family-wide or context-specific motifs, is more complicated. In order to exploit the full potential of the new sequence resources, it is clearly necessary to characterise precisely the conserved segments within these sequences.

### MSA program evaluation: alignment of locally conserved motifs

**Figure 6:**
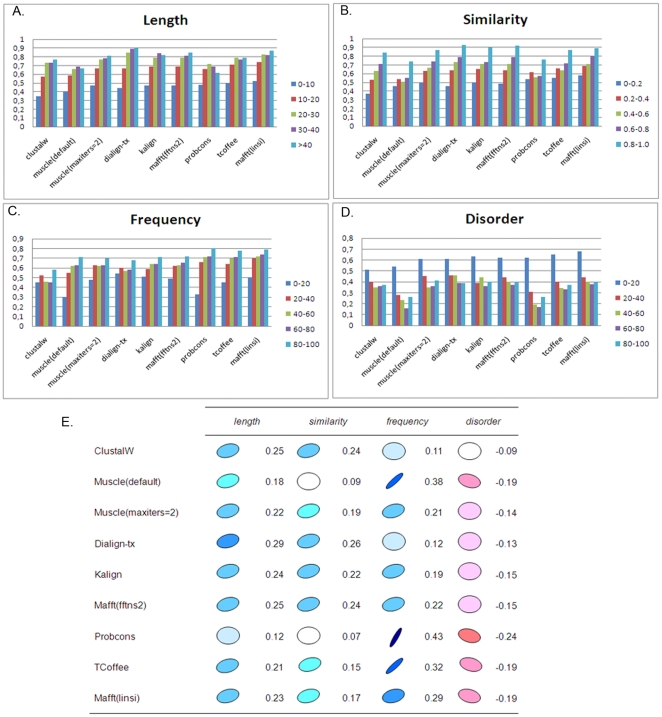
Factors affecting individual block alignment quality. Average block scores (BCS) for each MSA program and for each block attribute:(A) BCS versus similarity ( = 1-MD) of the sequences in the block, (B) BCS versus block length: average residue length of the block, (C) BCS versus frequency of occurrence of the block in the alignment, (D) BCS versus disorder: percentage of residues in natively disordered regions compared to folded domains.(E) Correlation of individual block scores (BCS)for each program with the various block attributes.

To investigate the ability of the MSA programs to identify context-specific or locally conserved motifs, we typified each individual block in the reference alignments using a number of different features: block length, sequence similarity in the block, the frequency with which the block is observed in thealignment, and the percentage of the block found in a natively disordered region. The alignment quality for each individual block was then measured using the Block Column Score (BCS) described in Methods. Figure 6(A–D) shows the distributions of the block scores obtained by each MSA program for each block attribute. BCSgenerally increased with increasing block length and increasing sequence similarity, as might be expected.Nevertheless, a correlation analysis (Figure 6A) showed that the programs did not respond in the same way to the different block features. For example, the scores obtained with the program Probcons were highly correlated with the frequency of the blocks, which implies that the blocks found in a small proportion of the sequences were aligned less well than those found in the majority of the sequences. In fact, for blocks found in less than 20% of the sequences, the mean BCS score for Probcons is 0.33, compared to 0.80 for blocks occurring in more than 80% of the sequences. This may be due to the probabilistic consistency-based objective function used in Probcons, which incorporates multiple sequence conservation information during the alignment of pairs of sequences. The defaultversion of Muscle and TCoffee were also affected by the frequency of the blocks. In the case of Muscle, this may be related to the iterative refinement stage, since the fast version with only 2 iterations was less sensitive. In contrast, Probcons and Muscle (default) were less sensitive than the other programs to the similarity of the blocks. The localization of the block in a natively disordered region had an adverse effect on the scores obtained by all the programs tested. Thus, blocks with more than 20% of the residues in natively disordered segments were aligned with BCS scores less than 0.5 by all aligners. This is in agreement with our original observation that most MSA programs available today are designed to align the globular, folded domains in proteins.

### Improving local alignment quality by combining methods

**Figure 7:**
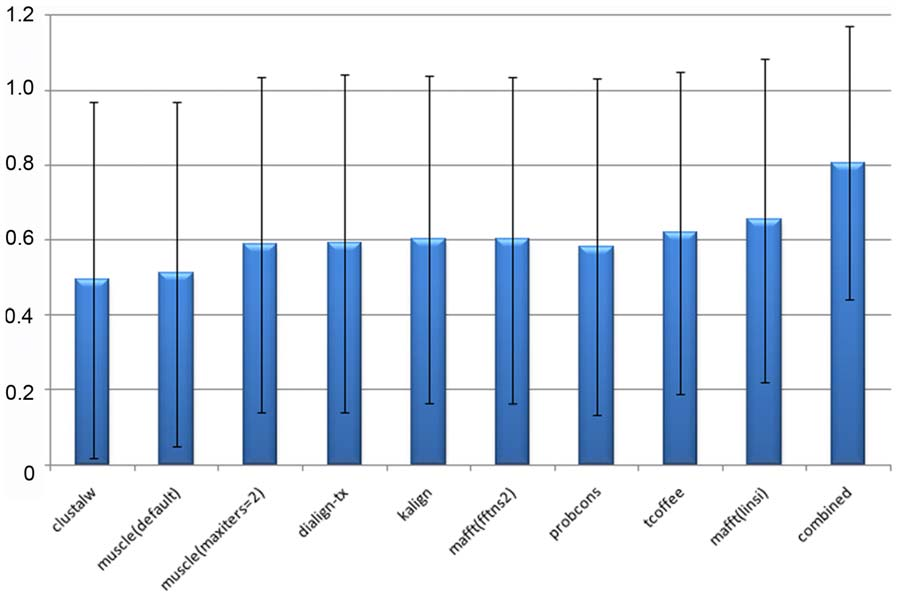
Comparison of block scores obtained by the different alignment programs. Mean block scores for the individual programs vary between 0.49 and 0.65. Combining the results from each program leads to an increased mean score of 0.81.Error bars correspond to one standard deviation. Asterisks indicate significant differences between the scores according to pairwise t-tests (significance level 0.05).

The experiments described above demonstrated some of the strengths and weaknesses of the different MSA construction methods. For a given set of sequences, different MSA programs often provide very different solutions, particularly outside the most conserved regions, as illustrated in Figure 1B. In order to test whether these differences could be exploited to improve local alignment accuracy, we determined a new score for each block, corresponding to the highest score obtained by any of the programs. This combined score was then compared to the block scores for each program individually (Figure 7). Of course, our combined score is a theoretical maximum, since it incorporates knowledge about the blocks from the reference alignment. Identifying *ab initio* conserved regions in different alignments and combining them in a single consensus alignment is more complicated. Nevertheless, the combined score represents a significant improvement over all the individual methods, with an increase in accuracy of almost 20%.

**Figure 8:**
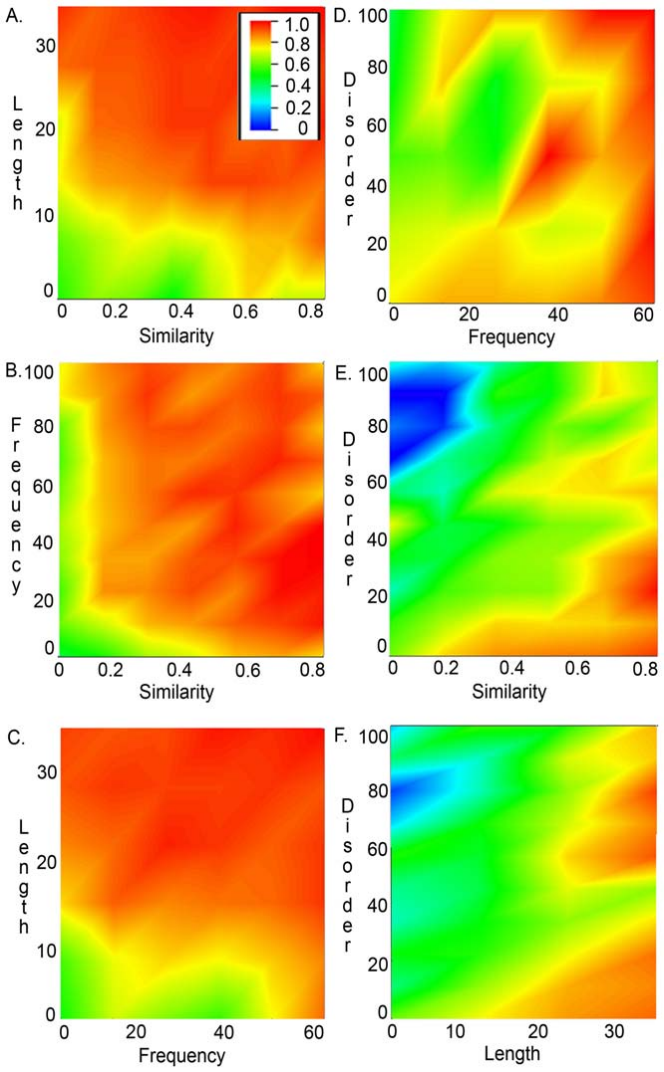
Alignability of blocks depends on various attributes. By combining 8 different MSA programs, a majority of blocks can be well aligned (red regions in the heat maps), but certain blocks remain problematic (blue, green regions). (A) Short blocks (<10 residues) with low similarity (<0.5) are aligned with 40–60% accuracy. (B) The frequency of occurrence in the alignment plays an important role. Blocks that occur in a majority of the sequences, even very divergent ones, are generally well aligned. (C) Short blocks (<10 residues) that occur in a majority of the sequences are also well aligned. (D to F) Blocks in natively disordered regions are generally less well aligned than those in folded regions, and short, divergent blocks are misaligned by all programs (blue regions).

Based on these combined scores, we posed the following question: can we model or predict the ‘alignability’ of specific blocks based on the attributes we have defined here? In other words, can we use these attributes to distinguish the blocks that can be aligned from those that cannot? The good news is that, by exploiting the individual capabilities of the recent algorithmic developments, a new milestone is attained where the globular domains present in a majority of the sequences can be accurately aligned (Figure 8). Even short blocks (<10 residues)with low similarity (<0.5) can be aligned with 40–60% accuracy (Figure 8A). However, the frequency of occurrence in the alignment plays an important role. Blocks that occur in a majority of the sequences, even very divergent ones, are generally well aligned (Figure 8B). Short blocks (<10 residues) that occur in a majority of the sequences are also well aligned (Figure 8C). Blocks in natively disordered regions are generally less well aligned than those in folded regions, and short, divergent blocks are misaligned by all programs (Figure 8D–F).

## Discussion

We have used a new alignment benchmark to investigate whether MSA programs are capable of constructing high quality alignments for the sequences resulting from modern biotechnologies. The overall alignment quality scores obtained by the different programs generally confirmed the trends observed in previous benchmark studies. One notable exception was the fact that increasing the number of sequences in the alignment did not lead to more accurate alignments on average. We hypothesize that this is due to the greater complexity of the large alignments, generally representing divergent protein families with complex domain organisations and an increased number of fragmentary and erroneous sequences.

A more detailed study of local alignment quality then allowed us to highlight a number of differences in the MSA methods tested. For example, for very divergent blocks, Mafft (linsi), TCoffee and Probcons were more successful. The local alignment method, Dialign-tx, and Kalign performed better for blocks that were conserved in small subsets of the sequences, while Mafft (linsi) achieved the highest scores for short blocks less than 10 residues long. Based on these results, we demonstrated that better alignment accuracy could be achieved by combining the strengths of the different programs. Unfortunately, the alignment accuracy still decreases when the domains are found less frequently in the alignment.In the future, new approaches will be needed to specifically address the problems of identifying the subfamily- or context-specific motifs and other blocks that occur less frequently in the alignment, and to handle the noise introduced by the numerousfragmentary and erroneous sequences.

There are a number of alternative solutions for coping with this additional complexity. First, assuming that the fragmentary and/or erroneous sequences can be identified, they can be excluded from the alignment, although this would discard a significant amount of information. Second, the missing or erroneous portions of the sequences can be predicted [41]. This however is difficult without the information from the alignment itself. Third, new algorithms and programs can be developed to handle the specific characteristics of the new sequences. Work in this direction has begun, with the development for example, of enhanced database searching algorithms such as CARMA [42], or MEGAN [43] that are more robust to the sequencing errors common in high throughput sequencing projects. In the MSA field, some aligners, such as Kalign, TCoffee or Probcons, provide estimators of local alignment accuracy that could be used to identify unreliable regions and eliminate them from subsequent analyses. The sensitivity/specificity of these accuracy scores has not been fully evaluated yet, although a comprehensive test could be performed using simulated sequences, where the true homology relationships between all sequence residues are known.

The alignment of blocks in the natively disordered regions is even more problematic. This is probably because the default parameters used in most MSA programs have been optimized on alignments of globular, folded domains, and most of the benchmarks used to evaluate the programs are based on structural superpositions of these domains. Although the 3D fold gives important clues to function, it does not represent the whole protein [38], [39]. The unstructured regions contain important regulatory signals, such as cellular localization or post-transcriptional modification sites, and many others waiting to be discovered. A number of groups have recently begun to develop new statistical models to represent many of these signals [44], [45] and it will be crucial to incorporate these models in future MSA programs.

So far, we have considered only the alignment of the conserved blocks that could be identified reliably, which cover less than 50% of the total alignment. The structural and/or functional roles of the remaining regions (shown in grey in Figure 1A) are still largely out of reach. We can draw parallels here with the evolving view of the human genome. When the genome was first sequenced, less than 5% of it was considered functional, the rest being ‘junk DNA’. Now, it is known that this so-called ‘dark matter’ does in fact contain numerous functional elements [46].

It is clear that the sequence alignment field now needs to evolve to cope with the challenges posed by the overwhelming flood of data. We have shown that the partitioning of the alignment into well characterised blocks allows a judicious combination of complementary methods resulting in more accurate alignments, particularly in the less well conserved regions. These alignments will in turn allow to highlight both conserved family signatures and specific regions that might suggest neo- or sub-functionalization, or other important genetic events. The next generation of MSA methods will undoubtedly incorporate other novel approachesthat will allow us to reveal the detailed picture of a gene's function and evolution in the context of their complex interaction and regulatory networks. We propose two major directions for future developments. First, the definition of alignment and block attributes opens the way to the exploitation of the latest developments in the field of statistical pattern recognition and data mining, aimed at extracting interesting or informative correlations (rules, regularities, patterns or constraints) from large data sets. Some recent research in this area has focused on the identification of rare patterns e.g.[47] and the problems of how to differentiate valid rare patterns from noise. Second, MSA algorithms can benefit from the new structural and functional “omics” data. In the same way that 2D and 3D structure information has already been used in methods such as 3D-COFFEE [26] or Refiner [27], or information from database homology searches in programs such as PRALINE[48]orPROMALS [49], other important data resources could be exploited to shed light on the unstructured and other ‘grey’ regions. For example, information about cellular localizationor specific molecular interactions could be used to guide the search for specific signals in these complex sequences.

Integration of these different algorithmic approaches and data types in knowledge-enabled, dynamic systems will ease and improve the complete MSA construction and analysis process; from the selection of a suitable set of sequences, via data cleaning and preprocessing, data mining and the evaluation of results, to the final knowledge presentation and visualization. Such systems could then be used to fully exploit the potential of MSAs as models of the underlying evolutionary processes that have created and fashioned extant genes and fine-tuned their structure, function and regulation.

## Materials and Methods

### Construction of reference alignments

**Figure 9:**
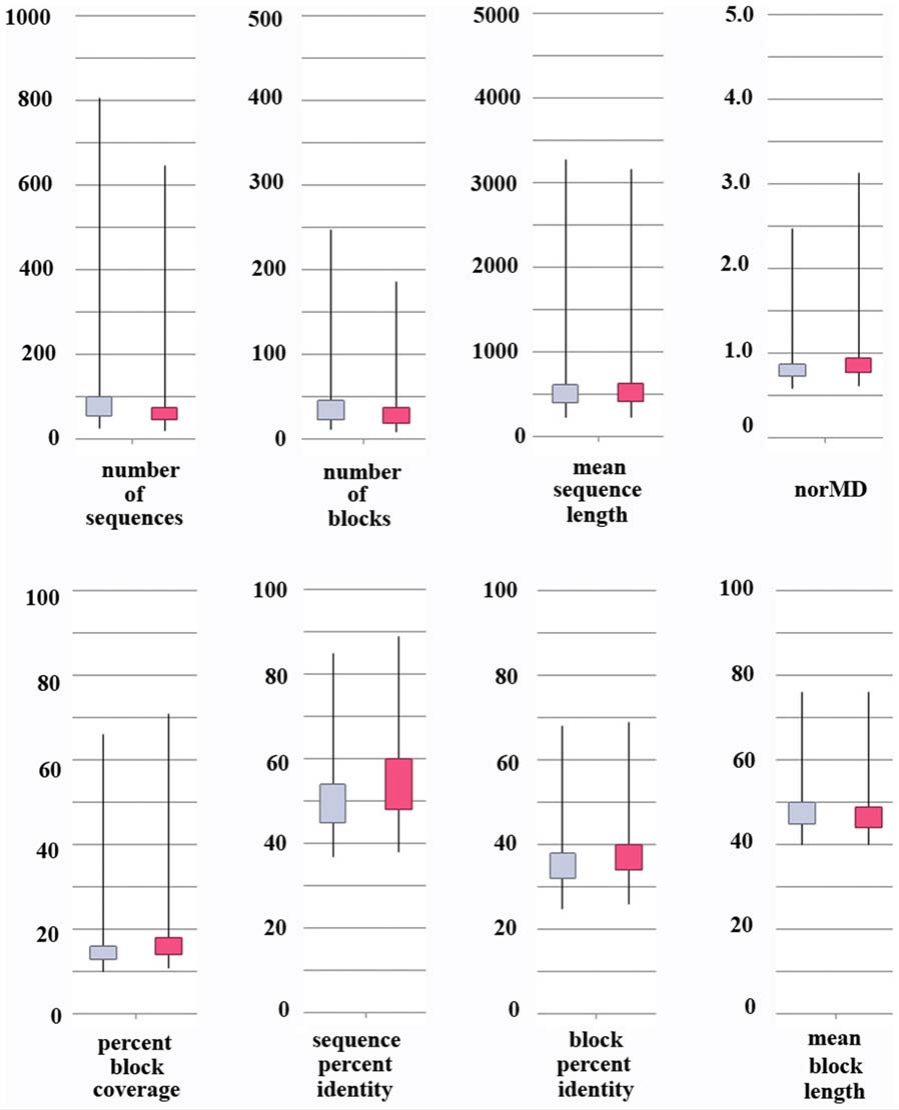
General statistics computed for the benchmark alignments. In the box-and-whisker plots, boxes indicate lower and upper quartiles, and whiskers represent minimum and maximum values. Blue boxes correspond to the alignment of all sequences. Red boxes correspond to the alignments containing only reliable sequences, with no identified sequence discrepancies.

The protein families used as benchmark test sets were selected to provide a variety of different multiple alignment problems (Figure 9). Thus, the number of sequences in each alignment ranges from 4 to 807. The mean sequence length for an alignment ranges from 56 to 3271 and mean residue percent identity ranges from 11 to 68. Detailed alignment statistics are available at ftp://ftp-igbmc.u-strasbg.fr/pub/msa_reference/stats.txt.

For each family, the reference alignment was constructed using a semi-automatic protocol similar to the one developed for the construction of the BAliBASE [50] alignment benchmark. Briefly, potential sequence homologs were detected by PSI-BLAST [51] searches in the Uniprot [52] and PDB [53] databases using a given query sequence. Of the 218 reference alignments, 122 (56%) have at least one sequence with known structure.Sequences with known 3D structure were then aligned using the SAP [54] 3D superposition program. Sequences with no known 3D structure were initially aligned by (i) identifying the most conserved segments in the PSI-BLAST HSP alignments with the Ballast [55] program and (ii) using these conserved segments as anchors for the progressive multiple alignment strategy implemented in DbClustal [56]. Unrelated sequences were removed from the multiple alignment using the LEON [57] program and the quality of the alignment was evaluated using the NorMD objective function. Finally, structural and functional annotations (including known domains from the Interpro database: www.ebi.ac.uk/interpro/) were added using the multiple alignment information management system (MACSIMS) [58].

The automatic alignment was then manually verified and refined to correct any badly aligned sequences or locally misaligned regions. The manual refinementincluded the alignment of known secondary structure elements and functional residues.At this stage, a subset of the complete set of sequences detected in the database searches was selected to ensure that the benchmark contains test sets of different sizes, thus representing a wide diversity of alignment problems. Alignments were edited with the JalView [59] editor which allows the user to visualize alignment conservation via various residue coloring schemes as well as conservation and consensus plots. The conserved regions were also explored according to the structural and functional information available for the sequence family.

### Alignment block calculation

For each reference alignment, blocks are defined that correspond to the reliably aligned regions, using the RASCAL [60] program. Briefly, the alignment is first divided horizontally into sequence subfamilies using Secator [61]. For each subfamily, sequence conservation is measured using the NorMD objective function in a sliding window analysis (window length = 5) along the length of the alignment. A block is then defined as a region in the alignment consisting of at least 3 columns, in which the NorMD is above the threshold value of 0.2. For each block in each subfamily, a profile [62] is built from the alignment and pairwise profile-profile comparisons are made to identify blocks shared between a number of subfamilies. This protocol is similar to the method used to identify blocks in the previous BAliBASE alignment benchmark [50], although in this case only regions conserved in all the sequences were marked as blocks.

This protocol led to the identification of 7985 blocks, representing on average 46% of the total multiple alignment (coverage ranged from <20% to >80%). The remainder of the sequence segments could not be aligned reliably based only on the sequences and structures present in the alignment. Thus, the blocks exclude local segments that are either (i) unalignable by sequence alone or (ii) not biologically alignable.

### Global alignment attributes

Four different attributes were calculated for each reference alignment, which reflect the overall difficulty of the alignment:

- the total number of sequences to be aligned,
- the average length of the sequences to be aligned,
- the norMD score which is an objective function for MSA based on the Mean Distance (MD) scores introduced in ClustalX [63]. A score for each column in the alignment is calculated using the concept of continuous sequence space introduced by Vingron and Sibbald [64] and the column scores are then summed over the full length of the alignment. The norMD scores also take into account the size of the alignment by calculating the maximum score attainable given the lengths of each of the unaligned sequences and assuming that the sequences are all identical.
- the percentage of the alignment covered by the blocks.

### Block attributes

Four different attributes were calculated for each block in each reference alignment:

- the average similarity of the sequence segments in the block is estimated using: Similarity = 1-MD, where MD = mean distance [40] of the sequences in the block,
- the length of the block, corresponding to the average number of residues for each sequence in the block,
- the frequency of occurrence of the block in the alignment, equal to the number of sequences in the block divided by the total number of sequences in the alignment,
- the structural context of the block, measured by the percentage of the residues in the block found in a predicted natively disordered (unstructured) region. Natively disordered segments were predicted using the IUPred program [65].

Although the benchmark test sets are designed to represent many different alignment problems, the sampling of the four attributes described here is not always homogeneous. For example, the test sets contain few blocks in disordered regions, which are also long or which occur frequently in the alignments. This results in some heterogeneity in the subsequent analyses, such as the results shown in figure 3 in the main text.

### Detection of sequence discrepancies

The sequences in the benchmark test sets were extracted from the public protein databases and may contain errors resulting from inaccurate gene structure prediction. Different types of prediction error were considered, such as excluding coding exons, including introns as part of the coding sequence, or wrongly predicting start and termination sites. We used the information in the reference multiple alignment to build a model of the protein family and sequences that deviated from this model were annotated as having potential sequence errors.

The sequences in the complete alignment were first divided into more related subfamilies using the Secator program [61]. Then, for each subfamily, sequences with discrepancies that might indicate errors in the corresponding gene structure, were identified using an empirical rule-based approach:

1. Badly predicted exons are identified using the RASCAL algorithm [60] as ‘outlier’ sequence segments. The method is summarized here and in Figure 2A. First, conserved ‘core blocks’ are identified for the subfamily, representing the sequence segments that are reliably aligned in the majority of the sequences within the subfamily. Then, for each core block, a weighted profile is built from the alignment and each sequence within the subfamily is assigned a score against the profile. Finally, a threshold score for each core block is defined based on the upper and lower quartiles of the sequence scores. Sequence segment outliers that score below the threshold are annotated as ‘discrepancies’ or potential errors.
2. Badly predicted start or stop sites are identified by considering the positions of the N/C-terminal residues for each sequence in the subfamily alignment (Figure 2B). For each sequence, the position of the terminal residue in the alignment is noted. A window, W, of ‘normal’ values is then determined, as follows: Q_1_-10<W<Q_3_+10, where Q_1_ and Q_3_ are the lower and upper quartiles respectively of the distribution of terminal positions. Sequences with terminal positions outside this window are annotated as potential deletion/extension errors.
3. Inserted introns (Figure 2C)are detected using the following rule: a potential inserted intron is detected if two subfamily alignment columns (i,j) exist such that ((n_i_ = N_i_) AND (n_j_ = N_j_) AND (N_k_ = 1 for i<k<j) AND (j-i> = 10)), where N_i_ is the total number of sequences in the subfamily (excluding fragments at column i), n_i_ is the number of residues in column i.
4. Missing exons (Figure 2D)are detected using the following rule: a potential missing exon is detected if two subfamily alignment columns (i,j) exist such that ((n_i_ = N_i_) AND (n_j_ = N_j_) AND (N_k_ = N-1 for i<k<j) AND (j-i> = 10)), where N_i_ is the total number of sequences in the subfamily (excluding fragments at column i), n_i_ is the number of residues in column i.

### Multiple alignment programs evaluated

The latest versions of 8 different multiple alignment programs (see below) were used to construct an alignment for each of the benchmark test sets. The programs were run using the default options for protein alignment, except for Mafft and Muscle. Mafft is a suite of programs offering various multiple alignment strategies, of which two complementary versions were tested: a rapid, less accurate version (fftns2) and an iterative refinement (linsi). For Muscle, two versions were tested: a fast, average accuracy version that limits the refinement to a maximum of 2 iterations (iters = 2), and the default options, which limits the refinement to a maximum of 16 iterations. The parallel version of TCoffee was run on 8 processors. Thus, a total of eight different versions of the alignment programs were tested (Table 1).

**Table 1:** Multiple sequence alignment programs used in this study.

Program	version	Availability
ClustalW[67]	2.0.12	www.clustal.org
Dialign-tx [68]	1.0.2	dialign-tx.gobics.de
Kalign [69]	2.03	msa.cgb.ki.se
Mafft (fftns2) [70]	6.815	align.bmr.kyushu-u.ac.jp/mafft/software
Mafft (linsi) [70]	6.815	align.bmr.kyushu-u.ac.jp/mafft/software
Muscle (iters = 2) [71]	3.8.31	www.drive5.com/muscle
Muscle (default) [71]	3.8.31	www.drive5.com/muscle
T-Coffee (parallel)[72]	8.99	www.tcoffee.org
Probcons [73]	1.12	probcons.stanford.edu

All programs were run on a Sun Enterprise V40z server (4 Opteron processors with 4×16 Gb memory) under RedHat Enterprise Linux.

### Evaluation procedure

#### Overall alignment quality scores

The alignments obtained from each of the 8 programs were compared to the corresponding reference alignments. Suppose we have a test alignment of N sequences and M blocks. For each block, b in the alignment containing n_b_ sequences and m_b_ columns, the i^th^ column of the block is assigned a score C_bi_ = 1 if all the residues in the column are aligned correctly, otherwise C_bi_ = 0. The score for each block ( =  C_bi_ averaged over its columns) is then weighted by the number of sequences in the block. The overall alignment quality, or Column Score (CS), is then:
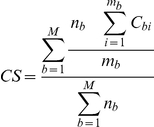



#### Block alignment quality scores

For each block, b in the alignment containing n_b_ sequences and m_b_ columns, the ith column of the block is again assigned a score C_bi_ = 1 if all the residues in the column are aligned correctly, otherwise C_bi_ = 0. The ability of the programs to align a specific block was estimated by calculating the block column score, (BCS)  =  mean column score in the block:
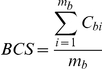
In this case, the block column scores are not weighted by the number of sequences in the block. Instead, each block has a maximum score of 1, regardless of the frequency with which it is observed in the alignment.

### Combining block alignment quality scores for different programs

For each reference alignment, a “combined score” was calculated corresponding to the maximal score possible if all correctly aligned blocks from each program were combined in a single alignment. For each block in the reference alignment, the maximum score obtained by any of the programs was selected and these maximal block scores were then averaged over the whole alignment.

### Availability

Unaligned sequences for all the reference alignments are available in FASTA format from ftp://ftp-igbmc.u-strasbg.fr/pub/msa_reference/msa_reference.tar.gz. The annotated alignments, including the block definitions, are provided in an XML format based on the MAO Multiple Alignment Ontology [66] and used by the MACSIMS systems [56]. The source code for the scoring schemes used here is available from ftp://ftp-igbmc.u-strasbg.fr/pub/msa_reference/bali_score_src_v4.tar.gz.
